# EEG microstate complexity for aiding early diagnosis of Alzheimer’s disease

**DOI:** 10.1038/s41598-020-74790-7

**Published:** 2020-10-19

**Authors:** Luke Tait, Francesco Tamagnini, George Stothart, Edoardo Barvas, Chiara Monaldini, Roberto Frusciante, Mirco Volpini, Susanna Guttmann, Elizabeth Coulthard, Jon T. Brown, Nina Kazanina, Marc Goodfellow

**Affiliations:** 1 Living Systems Institute, University of Exeter, Exeter, UK; 2 EPSRC Centre for Predictive Modelling in Healthcare, University of Exeter, Exeter, UK; 3 College of Engineering, Maths, and Physical Sciences, University of Exeter, Exeter, UK; 4 Cardiff University Brain Research Imaging Centre, School of Psychology, Cardiff University, Maindy Road, Cardiff, CF24 4HQ UK; 5 School of Pharmacy, University of Reading, Reading, UK; 6 University of Exeter Medical School, Exeter, UK; 7 Department of Psychology, University of Bath, Bath, UK; 8 San Marino Neurological Unit, San Marino Hospital, San Marino, Republic of San Marino; 9 Translational Health Sciences, University of Bristol, Bristol, UK; 10 School of Psychological Science, University of Bristol, Bristol, UK; 11 Centre for Biomedical Modelling and Analysis, University of Exeter, Exeter, UK

**Keywords:** Electroencephalography - EEG, Alzheimer's disease, Dementia, Biomarkers

## Abstract

The dynamics of the resting brain exhibit transitions between a small number of discrete networks, each remaining stable for tens to hundreds of milliseconds. These functional microstates are thought to be the building blocks of spontaneous consciousness. The electroencephalogram (EEG) is a useful tool for imaging microstates, and EEG microstate analysis can potentially give insight into altered brain dynamics underpinning cognitive impairment in disorders such as Alzheimer’s disease (AD). Since EEG is non-invasive and relatively inexpensive, EEG microstates have the potential to be useful clinical tools for aiding early diagnosis of AD. In this study, EEG was collected from two independent cohorts of probable AD and cognitively healthy control participants, and a cohort of mild cognitive impairment (MCI) patients with four-year clinical follow-up. The microstate associated with the frontoparietal working-memory/attention network was altered in AD due to parietal inactivation. Using a novel measure of complexity, we found microstate transitioning was slower and less complex in AD. When combined with a spectral EEG measure, microstate complexity could classify AD with sensitivity and specificity > 80%, which was tested on an independent cohort, and could predict progression from MCI to AD in a small preliminary test cohort of 11 participants. EEG microstates therefore have potential to be a non-invasive functional biomarker of AD.

## Introduction

Alzheimer’s disease (AD) is a neurological disorder in which progressive neurodegeneration and synaptic dysfunction result in impairments in a range of cognitive domains. Early diagnosis of AD has a wide range of clinical, social, and economic benefits^1–3^, but at present the definitive diagnosis of AD is made only post-mortem. Available tools for a diagnosis of probable AD in the clinic are mostly based on cognitive, biochemical, and neuroimaging markers^4^. Biochemical and neuroimaging techniques such as CSF or PET measurements of amyloid and tau have high sensitivity, but large multi-centre studies of CSF markers have shown specificity as low as 72% for prodromal diagnosis of AD^5^. The specificity of PET scans is higher, around 83–87%^6^.

Molecular and neuroimaging markers do not measure brain function, so combining them with functional markers is likely to further increase sensitivity and specificity of early diagnosis of AD. Neuropsychological evaluation assesses brain function, is non-invasive and relatively inexpensive, but is time consuming, subject to cultural and personal bias, and is unsuitable for prodromal diagnosis of AD in cognitively homogeneous cohorts of mild cognitive impairment (MCI) patients.

Electroencephalography (EEG) is a non-invasive measure of neuronal electrical activity in the brain. EEG is a promising diagnostic tool for disorders of the central nervous system, since it is low cost, non-invasive and currently implemented in healthcare systems around the world for diagnosis of epilepsy^7^. The first interest in using EEG as a diagnostic tool for AD was motivated by the associations between AD and epileptic activity^8^, but subsequent studies demonstrated limited success using epileptiform activity in the EEG to aid with the diagnosis of AD^9–11^. For this reason, focus has since shifted to EEG signature activities not directly related to network hyperexcitability, such as resting state power spectral and functional connectivity analyses^12^. At present, EEG testing is not routine in assessment of AD as the sensitivity and specificity is not sufficiently high, but commonly used EEG signatures of AD including spectral slowing and altered functional connectivity^12^ are typically assessed on a time scale of the order of seconds to minutes^13^, assuming stationarity over these epochs. Using a combination of measures containing complementary information may achieve greater classification accuracy^14,15^, therefore we hypothesise that combining these measures with analyses of non-stationarity within these epochs, novel information can be gained that can improve the ability of the EEG to classify AD^16,17^.

One such method is EEG microstate analysis, which involves studying the instantaneous topographic maps of the EEG^18^. Studies of EEG microstates have remarkably found the EEG to be comprised of only a small number of topographic classes^18^, such that the EEG remains stable in a given class for periods of the order tens or hundreds of milliseconds before rapidly transitioning to another class. These rapidly switching periods of quasi-stability are hypothesised to be the electrophysiological correlates of the rapid activation and inactivation of the brain’s resting state networks relating to different functions underpinning information processing^19–24^, earning microstates the nickname “atoms of thought”^24^. Thus, EEG microstates are a viable platform for exploring the changes in brain dynamics on the millisecond scale that may underpin impaired information processing and cognitive dysfunction in AD; indeed, alterations to microstates have been observed in healthy development and aging^25^ and a range of neurological disorders including frontotemporal dementia, schizophrenia, and depression^19^. This makes EEG microstate analysis a suitable candidate for biomarkers at a faster temporal scale than power spectral or functional network analysis.

Much interest has been given to how properties such as duration, coverage, and topography of microstates are altered in neurological disorders^19^. Alterations to patterns of transitions between classes have also been reported in neurological disease^26,27^, suggesting that studying transitioning behaviour of microstates may give further mechanistic insights into cognitive impairment in AD as well as increasing sensitivity of electrophysiological biomarkers. Indeed, recent studies have identified alterations to spatial patterns and temporal dynamics of EEG microstates in AD and MCI^17,26,28,29^. However, the ‘syntax analysis’^26,27^ used to analyse transitioning behaviour in these studies assumes the next microstate depends only on the present microstate (Markovian), and probabilities of transitions do not change over time (stationary). However, there is mounting evidence that microstate sequences are non-stationary and have non-Markovian transitioning properties^30–32^. Here, we present a novel analysis of the transitioning behaviour of EEG microstates, which does not rely on these assumptions, by applying the Lempel–Ziv complexity (LZC) algorithm^33^ to microstate sequences. LZC counts the number of unique sub-sequences within a sequence. Microstate sequences with low LZC are repetitive, exhibiting a limited number of transitioning patterns. Conversely high LZC is suggestive of complex transitioning. Because this measure does not make assumptions of Markovian transitioning, we hypothesise microstate LZC could aid with characterisation of the non-Markovian and non-stationary properties of temporal sequences of microstates^31,32^. Microstate LZC could potentially give new insights into alterations to information processing and transitioning between active networks in AD on the millisecond scale, and act as a more sensitive EEG-based neurophysiological signature of AD.

Here we explore microstate statistics in probable AD based on 20 s of resting state EEG, and the potential clinical applications of microstate LZC, combined with a power spectral measure^34^, for classification of early stage non-medicated probable AD patients. We then test this classifier on an independent set of EEG recordings acquired with standard 19 electrode systems (used in clinical settings) in medicated AD patients from a distinct geographical location. Finally, since a key limitation of many past studies is the lack of longitudinal data, we make a preliminary exploration of the combination of microstates LZC and spectral power to predict the conversion of MCI to AD, based on a small MCI cohort followed up longitudinally for up to 4 years.

## Methods

### Participants

Detailed descriptions of participant recruitment, inclusion criteria, diagnosis of probable AD, and analysis of demographics are given in Supplementary Material 1. AD patients (*n* = 21, 8 male) and amnestic MCI patients (*n* = 25, 16 male) were recruited from memory clinics in the South West of England (SWE) following clinical assessment on a consecutive incident patient basis. The diagnosis of probable AD was determined by clinical staff using neurological, neuroimaging, physical, and biochemical examination together with the results of family interview, neuropsychological, and daily living skills assessment according to DSM-IV^35^ and NINCDS-ADRDA guidelines^36^. Age matched cognitively healthy older adult (HOA) controls (*n* = 26, 14 male) were recruited from the memory clinics’ volunteer panels; they had normal general health with no evidence of a dementing or other neuropsychological disorder, according to NINCDS-ADRDA guidelines^36^. MCI participants who did not receive a dementia diagnosis within the four years following data acquisition were classified as stable (MCIs, *n* = 7, 5 male), those who had received an AD diagnosis were classified as converters (MCIc, *n* = 4, 4 male), and the remaining patients were excluded from the analysis (see Supplementary Material 1). All participants were free from medication known to affect cognition. Cognitive status at the time of data acquisition was quantified using the mini-mental state examination (MMSE)^37^.

A test cohort of AD patients (*n* = 9, 3 male) and HOA controls (*n* = 7, 4 male) was used to test generalizability of EEG biomarkers. Patients were recruited on a consecutive incident basis from the Republic of San Marino (RSM) database of dementia. The diagnosis of probable AD was determined according to the IWG-2 criteria^1^ by neurologists at the Republic of San Marino State Hospital. All AD patients underwent a neurological and functional examination, structural neuro-imaging (CT scan or MRI) and a full neuropsychological assessment. The inclusion criteria for the AD patients were a tau/Aβ_42_ ratio of > 0.52 at the CSF biochemical analysis and the presence of a significant episodic memory impairment as proved by a Memory Efficiency Index^38^ < 1.2. The exclusion criteria were evidence of vascular lesions (including lacunar infarcts and deep white matter changes) on structural neuroimaging and history or evidence of other neurological disorders or potential organic causes for memory disorders. Cognitively healthy controls were recruited from caregivers of patients and underwent a neurological examination and a neuropsychological assessment to exclude the presence of any cognitive disorder. In addition, all the AD patients from the San Marino cohort were being administered anticholinesterase drugs. Episodic memory impairment was quantified using the Rey Auditory Verbal Learning Test (RAVLT)^39^.

All participants provided written informed consent before participating and were free to withdraw at any time. All procedures were carried out in accordance with relevant guidelines and regulations; procedures were in accordance with the Declaration of Helsinki and approved by local ethics committees, i.e. the National Research Ethics Service Committee South West Bristol (Ref. 09/H0106/90) for the SWE cohorts and the Republic of San Marino Ethical Committee for Research and Experimentation (Ref. 0015 SM) and University of Exeter Medical School Research Ethics committee for the RSM cohort.

### EEG collection and pre-processing

20 s of resting-state, eyes-open EEG was used for all subjects. Data was sampled at 1000/512 Hz and recorded with 64/19 channels (SWE/RSM cohorts respectively). Data from both cohorts was pre-processed identically following previously described steps^34^. Details of the 20 s epoch selection and pre-processing can be found in Supplementary Material 2.

### Microstate analysis

#### Microstate extraction

Microstates were extracted using a *k*-means clustering method based on that of Koenig et al.^40^, using a *k*-means +  + algorithm used to select the initial *k* maps^41^, and 20 repetitions^40^. The Krzanowski-Lai criterion was used to assess the optimum number of microstates^42^. The median optimum over all subjects in the training set was *k* = 4, with no differences between groups. To ensure maps were comparable within cohorts (HOA or AD), a global clustering algorithm was performed^43^ for each cohort, and global maps between cohorts were aligned by calculating correlation coefficients of the maps and visual inspection. Additional details can be found in Supplementary Material 2.

#### Basic microstate statistics

Mean microstate duration for each class and percent time spent within a class (coverage) were calculated^19^. Analysis of microstate syntax, i.e. Markovian transitioning, was performed by extracting the transition matrix from the data by counting the number of transitions from class *i* to class *j* and normalizing by the total number of transitions^26,27^. For all measures, a two-way ANOVA was performed to assess statistical differences. Since ANOVA tests are parametric, significant results were post-hoc validated using non-parametric Mann–Whitney U tests. For all Mann–Whitney U tests, we report the *U*-statistic and its *z*-score (under the normal approximation)^44^ along side the *P* value as a measure of effect size. Topographic differences between groups were assessed with class-wise topographic analyses of variance (TANOVA)^23^ (Supplementary Material 2). The minimum possible *P*-value was *P* = 0.001, deemed sufficient to identify significant differences in topographies.

#### Microstate complexity

Here we present a novel measure of EEG microstate transitioning that, unlike the Markovian syntax analysis, does not rely on assumptions of stationarity or Markovianity^32^. The measure, referred to as *C*, involves calculating the Lempel–Ziv complexity (LZC)^33^ of the microstate transitioning sequence. The LZC of a string is defined as the number of different substrings within the string when read from left to right. A string is said to have low complexity if there are a small number of frequently repeating sequences. The algorithm for computing *C* is outlined in Supplementary Fig. S1 and Supplementary Material 3. LZC has in the past been used to calculate the complexity of a univariate EEG signal (Supplementary Material 3), but we believe use of this measure to explore the complexity of the microstate sequences is novel. All statistical comparisons of microstate LZC between groups was performed using non-parametric Mann–Whitney U tests.

## Results

### Participant demographics

Participant demographics, including age and neuropsychological scores, are outlined and analysed in Table 1, Supplementary Material 1and Supplementary Table S1–2. In both cohorts, AD patients demonstrated reduced neuropsychological test scores compared to cognitively healthy older adults (HOA). There were no differences in neuropsychological test score between MCI stable (MCIs) and MCI-to-AD converter (MCIc) at baseline.

**Table 1:** Data for healthy older adults and Alzheimer’s disease subjects for the training and test cohorts.

Cohort	HOA	AD	MCI (stable)	MCI (converter)
*South West of England cohort*				
Age (± SEM; years)	76 (± 7)	79 (± 9)	80 (± 2)	76 (± 6)
MMSE (± SEM; 0–30)	29 (± 1)	23 (± 3)	25 (± 1)	26 (± 1)
*N*	26	21	7	4
Male	14	8	5	4
Female	12	13	2	0
*San Marino cohort*				
Age (± SEM; years)	69 (± 2)	72 (± 2)		
RAVLT Immediate Recall (± SEM; 0–75)	40 (± 6)	20 (± 5)		
RAVLT Delayed Recall (± SEM; 0–15)	8.1(± 1.7)	.55(± .73)		
*N*	7	9		
Male	4	3		
Female	3	6		

### Analysis of microstates in Alzheimer’s disease

**Figure 1:**
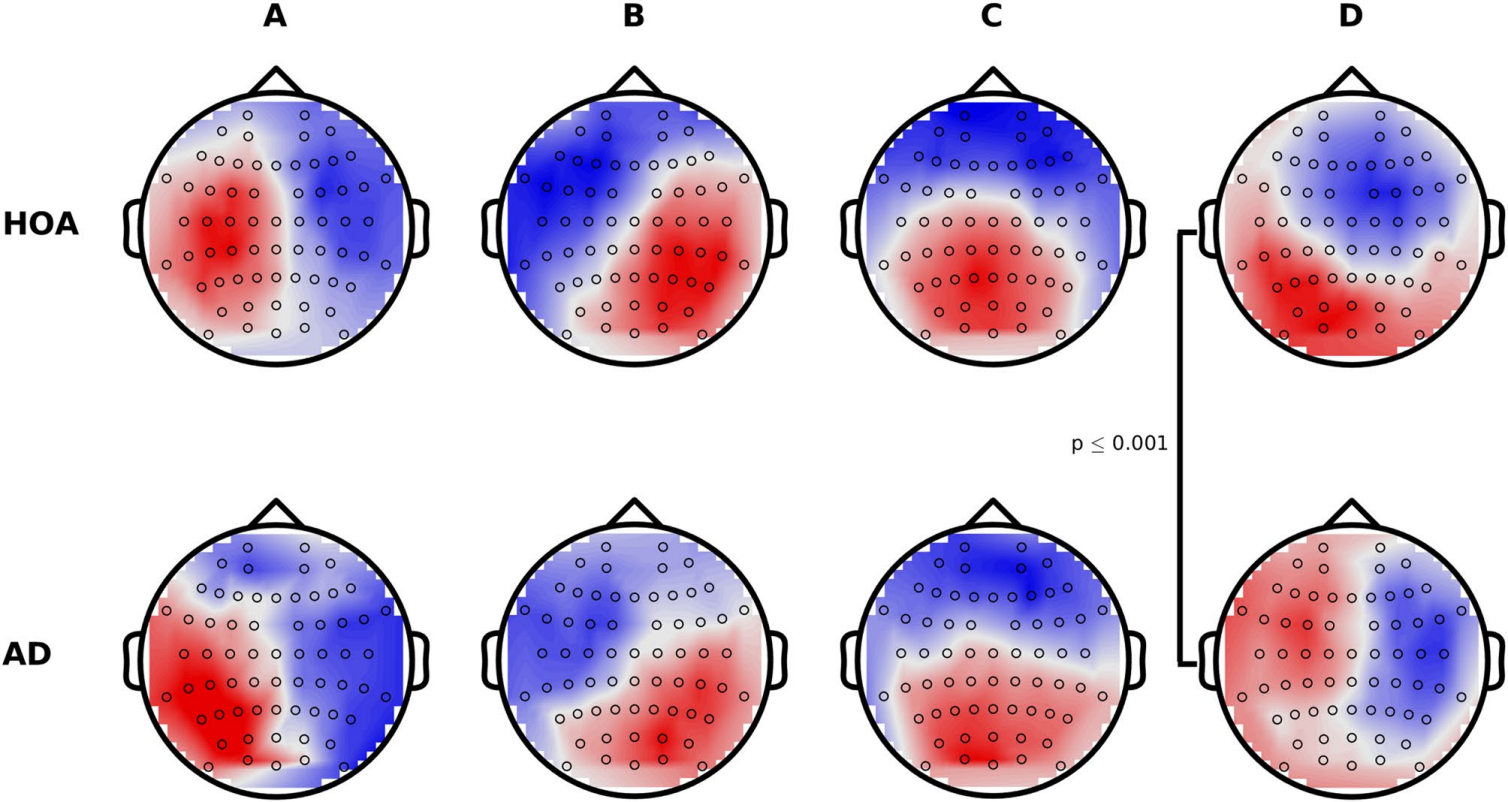
Microstate topographies for the four classes. (Top) Globally clustered maps for HOA cohort, for classes A-D from left to right. (Bottom) As above, but for the AD cohort. Black circles mark the electrode locations.

**Figure 2:**
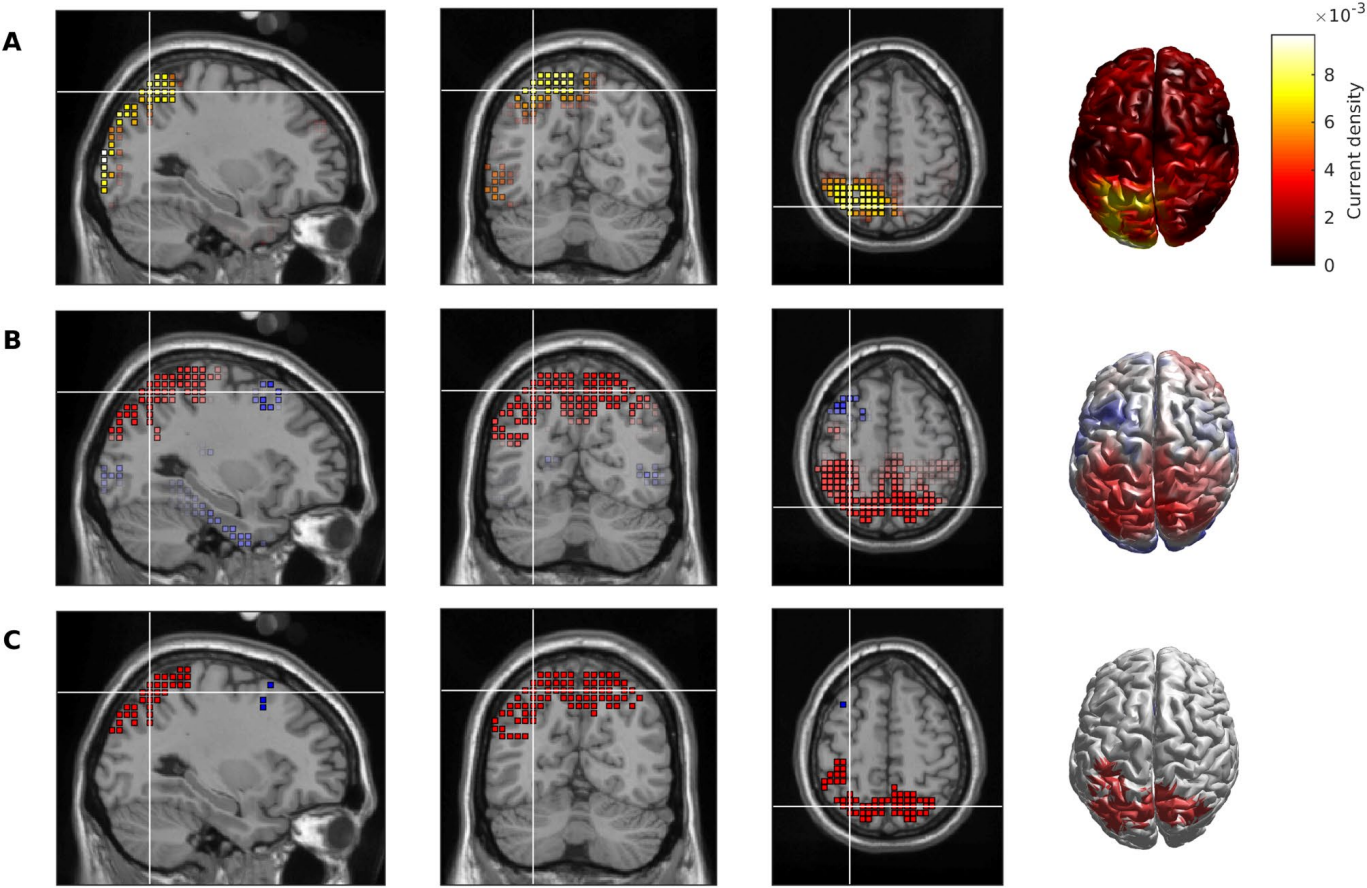
Cortical source generators underpinning alterations to microstate class D in AD. (**A**) Absolute value of the eLORETA solution to the instantaneous topography given by taking the difference between the global class D maps for HOA and AD. (**B**) *t*-statistic for voxel-wise comparisons of the subject-wise class D maps for HOA versus AD. Red indicates absolute value of current density is larger for HOA than AD, whilst blue is AD > HOA. (**C**) Voxels with *t*-values such that *P*  < .05. Red voxels indicate HOA > AD, and blue voxels are AD > HOA.

Topographies for each of the four microstate classes in the SWE HOA and AD data are shown in Fig. 1, and in the HOA subjects largely align with the classical microstate classes A-D associated with wakeful rest^18,19^, which are electrophysiological correlates of the auditory (A), visual (B), saliency (C), and frontopariental working memory/attention (D) resting state networks^18,21^. Class D was significantly altered in AD (*P* = 0.001; Fig. 1D). eLORETA was used to explore cortical generators underpinning this alteration (Supplementary Material 2). Figure 2A shows the eLORETA solution of the instantaneous map given by the difference of the global class D maps for HOA and AD. We subsequently calculated the eLORETA solution on a subject wise basis and calculated voxel-wise *t*-statistics to quantify spatially distributed differences in AD. Parietal sources were less activated in the AD subjects, particularly weighted more towards the left hemisphere (Fig. 2B,C).

**Figure 3:**
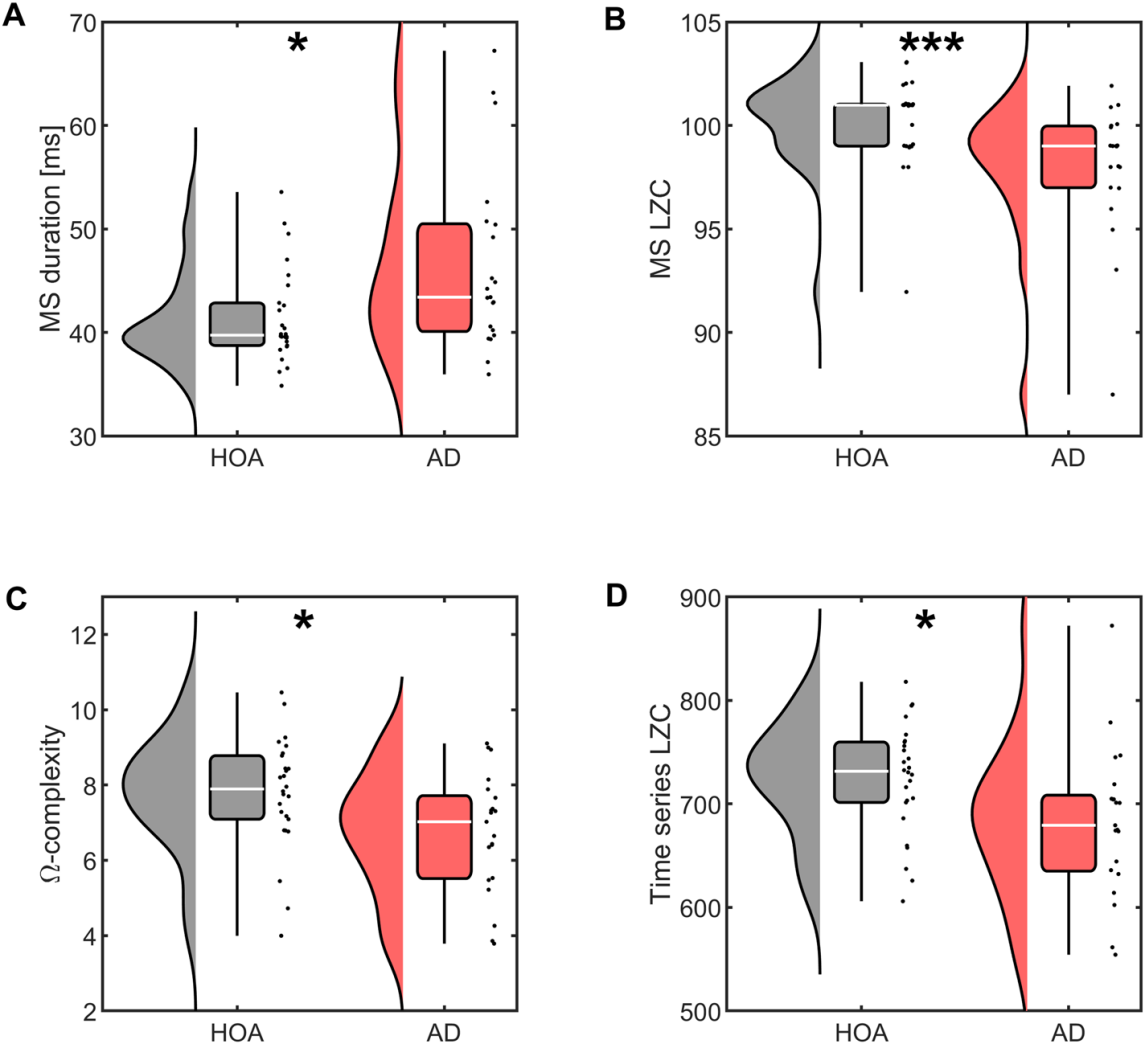
Microstate and complexity statistics are significantly altered in AD. (**A**) Mean duration of microstates. (**B**) Microstate LZC. (**C**) Ω-complexity. (**D**) Time series LZC. Descriptions of classical complexity measures C-D are given in Supplementary Material 3. Stars denote effect size of Mann–Whitney U test: *P < *.*05, **P < *.*01, ***P < *.*001. Points next to boxplots show values for each participant.

Having found changes to the topography and underlying cortical generators to class D in AD, we next explored whether the transitioning behaviour of the microstate sequences were altered. For each subject and microstate class, mean duration, coverage, and the Markovian syntax transition matrix were extracted. Supplementary Tables S3–S5 show the ANOVA tables for these tests. For mean duration, there was a significant disease group term (*F* = 9.95, *P* = *0.0*019), suggesting a significant increase in mean microstate duration in AD, averaged over all classes. We verified this significant increase in mean microstate duration with a non-parametric Mann–Whitney U test (*U* = *381, z* = *2.30, P* = *0.0*214, Fig. 3A). No significant results were found for coverage of microstates or Markovian transitioning. All analyses were additionally conducted on a pairwise basis for class D to further verify that changes to the topography of this class in AD did not alter transitioning statistics. No significant differences were found.

Studies have suggested that microstate transitioning is non-Markovian^30,32^. We therefore additionally calculated the Lempel–Ziv complexity (LZC) of the microstate transitioning sequence (*C*). *C* was significantly reduced in AD compared to HOA (*U* = *132.5, z* = −*3.05, P* = *0.0023*; Fig. 3B), suggesting that higher order or non-Markovian alterations to microstate transitioning are present in the EEG of AD patients, to which a Markovian syntax analysis is not sensitive. Additionally, this effect size was notably larger than other classical measures of EEG complexity used in past AD literature (Fig. 3C,D, Supplementary Material 3).

### Microstate complexity, combined with a single spectral feature, is a potentially useful biomarker of AD

Spectral slowing of the EEG in AD has been widely reported^12^, and has been identified as a powerful tool for classification of AD from the EEG^14,15^. A key aim of this study was to develop EEG microstate biomarkers for aiding diagnosis of AD, since we hypothesised that the difference in timescale between microstate measures and spectral measures would result in orthogonal information to increase classifier accuracy. Microstate LZC has a large effect size separating people with AD from controls and is uncorrelated with spectral slowing (Supplementary Material 3), so we next combined microstate LZC (*C*) with theta relative power (*θ*RP), a proxy for slowing previously identified in this dataset^34^ in a support vector machine (SVM) classifier (Supplementary Material 2).

**Figure 4:**
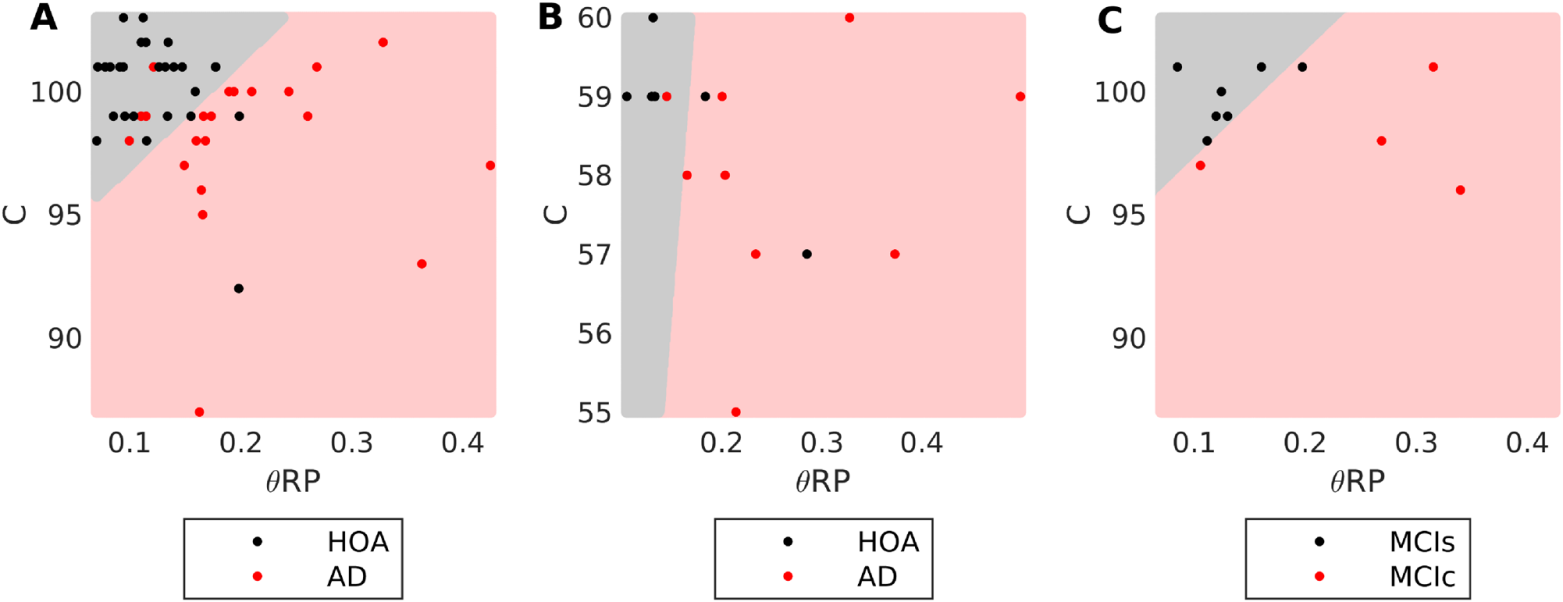
Separation of AD, HOA, and MCI using a SVM *θ*RP + *C* classifier. The SVM predictor classifies points within the pale red region as AD and points within the gray region as HOA. (**A**) Training data set overlaid on 64 channel model. (**B**) RSM data set overlaid on 19 channel classifier model. (**C**) MCI data set overlaid on 64 channel model. All models were trained on the data set shown in A. Blue circles in A show the support vectors for training in the 64 channel model.

Table 2 shows the results of ten fold cross validation of the classifier, trained on the 64 channel SWE EEG analysed above. When features were combined, classification rate (CR), sensitivity, and specificity were all greater than 80%, with a CR of 85.1%, demonstrating a notable improvement over the use of these features independently. Figure 4A shows this classifier in 2D (*θ*RP, *C*) space.

**Table 2:** Classification statistics from EEG measures in the training and test set.

Statistic (%)	SWE (HOA/AD)			RSM (HOA/AD)	MCIc/MCIs
	*C*	*θ*RP	*C* + *θ*RP	*C* + *θ*RP	*C* + *θ*RP
Classification rate	68.1	72.3	85.1	81.3	90.9
Sensitivity	61.9	66.7	81.0	88.9	100
Specificity	73.1	76.9	88.5	71.4	85.7

To test the classifier, the RSM cohort of clinical EEG was used as an independent test set. The SWE training data was spatially and temporally down-sampled to the same format as the RSM EEG, and the *θ*RP + *C* classifier re-trained on this down-sampled data set. Table 2 and Fig. 4B show the results of the classifier on the independent RSM test data. The CR of the test data was 81.3% (13/16 subjects), with sensitivity 88.9% (8/9 AD patients classified as AD by the model) and specificity 71.4% (5/7 HOA subjects classified as HOA by the model). These results suggest the SVM classifier may be generalizable to new clinical data sets and is not due to overfitting to our data. However, to confirm this result a larger test dataset will be necessary.

### Microstate complexity and slowing have predictive power for diagnosing prodromal AD

Finally, to test whether the classifier presented here may be useful as a tool for prodromal diagnosis of AD^1^, i.e. predicting whether mild cognitive impairment (MCI) is due to prodromal AD, EEG recorded from a test set of MCI patients was classified by the model. Subjects were classified as MCIs or MCIc based on their clinical diagnosis four years following data acquisition. At the time of data acquisition, there was no difference in clinical diagnosis or MMSE scores for the subjects (Table 1, “Participant demographics” section).

Results of the classification are shown in Fig. 4C and Table 2. In this preliminary cohort, the model had a classification rate of 90.9%, correctly predicting the four-year diagnosis of 10/11 MCI patients. One MCIs subject was incorrectly classified as a converter.

## Discussion

The aim of this study was to explore microstate analysis as a predictive tool for the diagnosis of probable AD. A classifier was trained on a cohort of people with probable AD and cognitively healthy older adults recruited from the South West of England (SWE), using 64-channel EEG. Subsequently, the classifier was tested on an independent cohort recruited from the Republic of San Marino (RSM), using a standard 19-channel EEG machine for clinical use. Whilst the RSM AD population was treated with acetylcholinesterase (AChE) inhibitors, the SWE cohort was unmedicated. This is an important fact in support of this method for early diagnosis, as the use of AChE inhibitors to treat people with AD is very common and a possible confounding factor for functional measures. The accuracy of our method was measured against a clinical diagnosis of probable AD based on IWG-2^1^ (test data) or DSM-IV^35^ and NINCDS-ADRDA guidelines^36^ (training data).

### Microstate complexity measure

In this work we present a novel application of Lempel–Ziv complexity (LZC)^33^ to microstate EEG sequences for the diagnosis of AD. LZC was chosen to study microstate transitions over classical Markovian syntax analysis since microstate transitioning is neither Markovian nor stationary^30,32^. We found that the LZC of microstate transition sequences had a notably larger effect size in separating AD patients from controls than Markovian syntax analysis, suggesting higher order alterations to transitioning between microstates in AD.

As a multivariate measure, microstate LZC additionally captures both spatial and temporal complexity, resulting in a larger effect size than previously reported measures of EEG complexity (Supplementary Material 3). A closely related univariate measure of EEG complexity is time series LZC, which has in the past been used to identify reduced complexity of the EEG in patients with AD by binarizing a univariate EEG time series based on a threshold and then calculating the LZC of this binary sequence (Supplementary Material 3). Application of LZC to the microstate sequences is an improvement over this method for several reasons. By using microstates, binning of the EEG is chosen based on repeating spatiotemporal patterns which have some neurophysiological basis related to active networks^18,23^, as opposed to an arbitrary threshold. Furthermore, microstate LZC accounts for the multivariate nature of the EEG, not accounted for in classical time series LZC. Microstate LZC is a spatially extended version of the LZC method classically used in EEG literature. Finally, unlike time series LZC^45^, microstate LZC is uncorrelated with EEG slowing (Supplementary Material 3), meaning it is a useful EEG biomarker of AD when combined with slowing.

### Alterations to class D and the frontoparietal network

The topography of microstate class D was altered in AD. Previous studies give conflicting reports on alterations to microstate topographies in AD; in agreement with our results, Smailovic et al.^29^ found alterations to the topography of class D in AD but also found alterations to class A, while Schumacher et al.^28^ found alterations to all classes in AD and Nishida et al.^26^ found alterations to none. Differences in the number of channels is unlikely to explain these inconsistencies in results, as microstates are reliable with 8 or more channels^43^. The use of eyes-open data is also an unlikely to give inconsistent results^46^ (Supplementary Material 4). However, methodological differences may potentially explain opposing findings; in Nishida et al.^26^ all participants were used for defining the topographies of the four classes, in Smailovic et al.^29^ only the controls were used to define the classes, whilst in our study and that of Schumacher et al.^28^ classes were defined independently for each cohort. Additionally, Schumacher et al.^28^ used five microstate classes in their analysis, while the other studies discussed here used four.

It has been suggested that class D is related to the frontoparietal network and the attention and working memory cognitive domains^19,21,26,46^. Reduced parietal activation underpinned the change in topography of class D in AD, supporting the hypothesis of dysfunction of the frontoparietal network. A possible mechanism for this is disrupted frontoparietal white matter integrity, since altered frontoparietal functional and effective connectivity have been reported in recent fMRI studies of AD^47,48^.

### Alterations to microstate duration and transitioning statistics

In this study, mean microstate duration was found to increase in AD. Reports of altered microstate duration are inconsistent in the past literature, which has found increased^28,29,49^, decreased^16,50,51^, or unchanged^17,26^ durations in AD. Of these studies, four have used modern clustering methods comparable to this study. Two of these studies identified no differences in duration^17,26^. Eyes-open versus eyes-closed EEG affects microstate duration (Supplementary Material 4)^46^, potentially explaining our conflicting results. Conversely, Smailovic et al.^29^ used modern clustering methods to study microstates from large cohorts of several hundred participants, and found increased mean duration in line with our results. Schumacher et al.^28^ also reported increased mean duration of microstates in AD in a smaller cohort. Interestingly, we might expect microstate duration to increase in AD due to slowing of the EEG^32^ which is well established in AD^12^—this was the original hypothesis made by Dierks et al.^16^, who expressed their surprise that a decreased microstate duration was found instead. Increased duration and reduced microstate LZC are suggestive of slower and more repetitive transitioning between active networks in AD, possibly indicating less complex information processing and giving insight into the mechanisms underpinning cognitive deficits in AD.

### Limitations

There are some limitations to our study. A primary limitation is the sample size. Our training cohort from the south west of England consisted of 21 probable AD patients and 26 cognitively healthy controls. For a mechanistic study into microstate statistics, these numbers are consistent with other recent studies in the field^17,26,28^. Many of the results presented here (increased microstate duration, altered topography of class D) were consistent with those of Smailovic et al.^29^, whose study consisted of several hundred participants, providing evidence to support that our results are unlikely to be of small sample size. However, it should be noted that Smailovic et al.^29^ also identified alterations to class A while our study did not, potentially as a result of lower power due to smaller sample size in our study. For clinical utility, the classifier should work on the individual level. Ultimately, larger cohorts will be required to quantify efficacy of the classifier. One strength of the study was the promising initial results from testing the classifier on an independent cohort and a cohort of people with MCI, a step which is not often done, but it should be noted that these sample sizes were even smaller (9 AD, 7 controls, 11 MCI). Hence, the work presented here should be viewed as a preliminary study, and future work will involve further testing of these results in additional cohorts, particularly with respect to prediction of conversion from MCI.

A second limitation relates to the diagnostic criteria for participants. In the primary cohort of training data (and the MCI test data), diagnosis of AD was based on the DSM-IV^35^ and NINCDS-ADRDA criteria^36^. Dubois et al.^52^ reviewed the limitations of these criteria, the foremost of which is limited specificity against other forms of dementia. In this study, these guidelines were used alongside neuroimaging and biochemical examination to provide *in-vivo* evidence of AD pathology, which is likely to improve specificity^52^; nonetheless, at most here we can describe our participants as having “probable AD”, and results should be interpreted as such. The IWG-II criteria^1^ are recent criteria for diagnosis of AD based on both clinical phenotype and *in-vivo* evidence of AD pathologies, which were used to diagnose AD in our test data. This inconsistency in diagnostic criteria between cohorts is a potential biasing factor for the study which should be explored further in future studies.

In addition to diagnostic criteria, several other factors differ between cohorts, for example medication and age. This is both a limitation and potential strength of the study; while differences between cohorts may reduce the power of the study, the results presented here suggest the classifier may be robust to these factors.

## Conclusions

In this work we assessed microstate analysis, in combination with power spectral analysis, as a low-cost, non-invasive tool for aiding the diagnosis of AD. Additionally, our observations can provide crucial insight into the mechanisms underpinning cognitive impairment in AD. Alterations to the frontoparietal network (namely parietal inactivation) was shown to relate to a changing topography in microstate class D. This network and microstate class are related to attention and working memory^21,26,46^, which are impaired early in the AD staging^53^. Microstate duration was found to increase in AD, whilst a novel application of Lempel–Ziv complexity to the microstate transitioning found decreased complexity in AD. These results are suggestive of slower and more repetitive microstate transitions, which potentially reflects similar attributes to the transitioning between active brain networks associated with a range of cognitive domains^19–24^. Preliminary data suggest use of microstate complexity as a biomarker which can potentially aid with early diagnosis and prediction of future conversion to AD. Whilst medication is known to alter microstate statistics^54,55^, our classifier retained high classification statistics for a probable AD diagnosis in an independent, medicated cohort.. The mechanistic insights presented in this study, paired to future, preclinical characterization of cellular correlates of microstate alterations in transgenic rodent models of AD pathologies may aid future drug development and more accurate diagnostic and prognostic tools for AD.

## Supplementary information

Supplementary information.
